# Lack of association between atopic dermatitis and COVID-19 severity: results from a case-control study

**DOI:** 10.17179/excli2025-8197

**Published:** 2025-05-22

**Authors:** Martha Débora Lira Tenório, Pedro Dantas Oliveira, Paulo Ricardo Martins-Filho

**Affiliations:** 1 Graduate Program in Health Sciences, Federal University of Sergipe, Sergipe, Brazil; 2 Investigative Pathology Laboratory, Federal University of Sergipe, Sergipe, Brazil; 3 Department of Medicine, Federal University of Sergipe, Aracaju, SE, Brazil

## ⁯

The potential association between atopic dermatitis (AD) and COVID-19 has garnered increasing interest in dermatology. AD, a prevalent chronic inflammatory disease, is characterized by skin barrier dysfunction and immune dysregulation, which may heighten susceptibility to infections. Despite several clinical and epidemiological studies investigating the risk of SARS-CoV-2 infection and COVID-19 severity in AD patients, results remain inconsistent due to confounding factors (Fan et al., 2022[4]). This study aimed to evaluate the association between AD and COVID-19 severity, thereby clarifying whether this inflammatory skin disorder affects the clinical course of SARS-CoV-2 infection.

We conducted a case-control study at a tertiary center in Sergipe, Northeastern Brazil, prior to the introduction of the national COVID-19 vaccination program. The study included patients with confirmed SARS-CoV-2 infection by polymerase chain reaction (PCR) who sought medical care at our hospital between April 2020 and December 2021. Cases were defined as patients with severe or critical COVID-19 requiring admission to an intensive care unit (ICU), whereas controls had mild COVID-19 without viral pneumonia or hypoxia. Controls were advised to follow standard public health measures, including hand hygiene, face mask use, and home isolation. Medications were prescribed based on symptoms, with telephone follow-ups every 24 hours for 14 days after symptom onset and in-person evaluations provided as needed. Exclusion criteria included death, immunodeficiency, incomplete data, age under 18 years, and loss to follow-up. Patients with moderate COVID-19, characterized by fever, cough, dyspnea, and tachypnea but without severe pneumonia (SpO2 ≥ 90% on room air), were also excluded to improve the precision of clinical severity classification given the variability in moderate cases.

AD symptoms were assessed using the International Study of Asthma and Allergies in Childhood (ISAAC) questionnaire (Asher et al., 1995[2]; Solé et al., 1998[10]). Data collection occurred via telephone interviews after hospital discharge for cases and during home isolation for controls. To avoid confusion between COVID-19-related symptoms and preexisting conditions, the questionnaire focused on health status and symptoms experienced prior to COVID-19 onset. This ensured consistency in data collection while minimizing additional exposure risks. Furthermore, we previously demonstrated in this same population that asthma was associated with COVID-19 severity, whereas rhinitis presented a protective effect (Lira Tenório et al., 2024[8]). For this study, we focused exclusively on AD patients to minimize confounding with these inflammatory airway diseases. Age, sex, comorbidities (hypertension/diabetes mellitus), drug allergies, and chronic use of antihypertensives, antidiabetic agents, beta-2 agonists, antihistamines, and corticosteroids were considered as adjustment variables. Statistical analyses included univariate logistic regression, followed by multivariate analysis with backward variable selection. Multicollinearity was assessed using the Variance Inflation Factor (VIF). Results were expressed as odds ratios (OR) with 95% confidence intervals (CI). Analyses were performed using R software (R Foundation for Statistical Computing, Vienna, Austria). 

A total of 122 patients were included in the study, of whom 61 had severe or critical COVID-19 (case group) and 61 had mild COVID-19 (control group). The mean age was 45.2 years (±16.3), with 25 participants (20.5%) aged 60 or older. Females comprised 51.6% of the sample (n = 63). Hypertension and/or diabetes were reported in 34.4% (n = 42), chronic medication used in 45.1% (n = 55), and drug allergy in 13.9% (n = 17). Eight participants (6.6%) had a diagnosis of AD, including 6 (9.8%) in the case group and 2 (3.3%) in the control group. Significant associations were found for male sex (OR = 5.5; 95% CI: 2.3-13.6; p < 0.001), age ≥ 60 years (OR = 4.6; 95% CI: 1.2-16.8; p = 0.022), and comorbidities (OR = 5.3; 95% CI: 1.9-14.3; p = 0.001) with severe/critical COVID-19. No significant association was observed between AD and COVID-19 severity (OR = 4.8; 95% CI: 0.8-29.4; p = 0.091) (Table 1; supplementary information).

The results of the present study did not identify a significant association between AD and COVID-19 severity, aligning with previous research that also found no causal relationship between AD and severe COVID-19 outcomes, including hospitalization, the need for oxygen therapy, and mortality (Rakita et al., 2021[9]; Lin and Hong, 2022[7]). These findings reinforce the hypothesis that AD, despite being a chronic inflammatory skin disease, may not have a significant systemic impact on SARS-CoV-2 infection. Although AD is characterized by a predominant Th2 immune response, mediated by cytokines such as interleukin-4 (IL-4) and interleukin-13 (IL-13), which are hypothesized to reduce the expression of the ACE2 receptor on airway epithelial cells and potentially hinder viral entry (Kimura et al., 2020[5]), this theoretical protective effect does not appear to translate consistently into a reduction in the COVID-19 severity among AD patients (Chen et al., 2024[3]).

The phenotypic and endotypic heterogeneity of AD may partly explain this lack of association. While Th2 immune responses are central in many cases of AD, not all patients exhibit this profile. A significant subgroup demonstrates non-atopic characteristics, such as low IgE sensitization and reduced IL-13 levels, potentially mitigating the impact of Th2-driven inflammation on susceptibility or progression of COVID-19 (Leyva-Castillo et al., 2020[6]; Abuabara and Langan, 2023[1]). Consequently, the influence of AD on COVID-19 may vary based on the specific phenotype of the condition. External factors may also act as confounders. Immunomodulatory medications commonly used in AD treatment may alter immune responses to SARS-CoV-2, though their impact on COVID-19 outcomes remains unclear. Furthermore, the chronic nature of AD often necessitates frequent healthcare visits, increasing the likelihood of early COVID-19 diagnosis. Combined with heightened awareness of hygiene and prevention practices, these factors may influence the observed clinical outcomes (Fan et al., 2022[4]).

In conclusion, our findings do not support the hypothesis that AD influences COVID-19 severity. Further research should explore the differences among AD phenotypes and their potential impact on viral outcomes to clarify this relationship.

## Declaration

### Ethics

The study was approved by the Research Ethics Committee of Federal University of Sergipe (CAAE 31079720.5.0000.5546).

### Funding

None. 

### Conflicts of interest

None. 

### Acknowledgments

PRMF is a research productivity fellow at the National Council for Scientific and Technological Development (CNPq), Brazil.

### Authors contributions

MDLT: Conceptualization, Methodology, Investigation, Formal analysis, Writing - original draft, Writing - review & editing; R.S.M.: Conceptualization, Methodology, Investigation, Writing - review & editing; PDO: Conceptualization, Project administration, Methodology, Writing - review & editing; PRMF.: Conceptualization, Project administration, Methodology, Formal analysis, Writing - original draft, Writing - review & editing.

## Supplementary Material

Suppl. information
