# Supplementary material for: Lack of association between atopic dermatitis and COVID-19 severity: results from a case-control study
Source: EXCLI J. 2025 May 22;24:558–61. doi: 10.17179/excli2025-8197 (PMC12127519; doi:10.17179/excli2025-8197)
Supplement: Suppl. information [file EXCLI-24-558-s-001.pdf]

EXCLI Journal 2025;24:558-561 – ISSN 1611-2156
Received: January 17, 2025, accepted: April 17, 2025, published: May 22, 2025

Supplementary information to:

Letter to the editor:

LACK OF ASSOCIATION BETWEEN ATOPIC DERMATITIS AND COVID-19 SEVERITY: RESULTS FROM A CASE-CONTROL STUDY

Martha Débora Lira Tenório[1,2], Pedro Dantas Oliveira[3], Paulo Ricardo Martins-Filho[1,2,*]

[1] Graduate Program in Health Sciences, Federal University of Sergipe, Sergipe, Brazil
[2] Investigative Pathology Laboratory, Federal University of Sergipe, Sergipe, Brazil
[3] Department of Medicine, Federal University of Sergipe, Aracaju, SE, Brazil

***Corresponding author**: Paulo Ricardo Martins-Filho, Universidade Federal de Sergipe, Hospital Universitário, Laboratório de Patologia Investigativa.
Rua Cláudio Batista, s/n. Sanatório. Aracaju, Sergipe, Brazil. CEP: 49060-100. E-mail: prmartinsfh@gmail.com

https://dx.doi.org/10.17179/excli2025-8197

This is an Open Access article distributed under the terms of the Creative Commons Attribution License (http://creativecommons.org/licenses/by/4.0/).

EXCLI Journal 2025;24:558-561 – ISSN 1611-2156
Received: January 17, 2025, accepted: April 17, 2025, published: May 22, 2025

Table 1: Association Between Demographic and Clinical Variables and COVID-19 Severity

Variables	Total (n=122)	COVID-19		Univariate Analysis		Multivariate Analysis	
		Mild (n=61)	Severe/Critical (n=61)	OR (95% CI)	p-value	OR (95% CI)	p-value
**Sex**							
Female	63	43 (70.5%)	20 (32.8%)				
Male	59	18 (29.5%)	41 (67.2%)	4.9 (2.3 – 10.6)	< 0.001	5.5 (2.3 – 13.6)	< 0.001
**Age**							
< 60 years	97	57 (93.4%)	40 (65.6%)				
≥ 60 years	25	4 (6.6%)	21 (34.4%)	7.5 (2.4 – 23.5)	< 0.001	4.6 (1.2 – 16.8)	0.022
**Comorbidities**							
No	80	51 (83.6%)	29 (47.5%)				
Yes	42	10 (16.4%)	32 (52.5%)	5.6 (2.4 – 13.1)	< 0.001	5.3 (1.9 – 14.3)	0.001
**Drug allergies**							
No	105	51 (83.6%)	54 (88.5%)				
Yes	17	10 (16.4%)	7 (11.5%)	0.7 (0.2 – 1.9)	0.435		
**Chronic medication use**							
No	67	39 (63.9%)	28 (45.9%)				
Yes	55	22 (36.1%)	33 (54.1%)	2.1 (1.0 – 4.3)	0.047		
**Atopic dermatitis**							
No	114	59 (96.7%)	55 (90.2%)				
Yes	8	2 (3.3%)	6 (9.8%)	3.2 (0.6 – 16.6)	0.163	4.8 (0.8 – 29.4)	0.091

OR, odds ratio; CI, confidence interval.
